# Knowledge, attitudes and practices regarding HIV/AIDS among male high school students in Lao People's Democratic Republic

**DOI:** 10.7448/IAS.16.1.17387

**Published:** 2013-03-11

**Authors:** Bounbouly Thanavanh, Md. Harun-Or-Rashid, Hideki Kasuya, Junichi Sakamoto

**Affiliations:** 1 Public Health Department, Nalao, Sayaboury, Lao PDR; 2 Young Leaders’ Program in Healthcare Administration, Nagoya University Graduate School of Medicine, Nagoya, Japan; 3 Department of Surgery II, Nagoya University Graduate School of Medicine, Nagoya, Japan

**Keywords:** knowledge, attitudes, practices, HIV/AIDS, Lao PDR

## Abstract

**Introduction:**

Inadequate knowledge, negative attitudes and risky practices are major hindrances to preventing the spread of HIV. This study aimed to assess HIV-related knowledge, attitudes and practices (KAPs) of high school students in Lao People's Democratic Republic (PDR).

**Methods:**

A cross-sectional study on unmarried male students aged between 16 and 19 years old was undertaken in 2010 to evaluate their KAPs. We selected 300 eligible grade VII students through systematic random sampling from different high schools in one province of Lao PDR.

**Results:**

The majority of students surveyed were aware that HIV can be transmitted by sexual intercourse (97.7%), from mother to child (88.3%) and through sharing needles or syringes (92.0%). Misconceptions about transmission of HIV were observed among 59.3% to 74.3% of respondents. Positive attitudes towards HIV/AIDS were observed among 55.7% of respondents. Nearly half of the surveyed students (45.3%) said that they would be willing to continue studying in a school with HIV-positive friends, and 124 (41.3%) said they would continue attending a school with HIV-positive teachers. Ninety-four (31.3%) students had a history of sexual intercourse, and 70.2% of these students had used a condom. However, only 43.9% said they used condoms consistently. Students with medium and high levels of knowledge were 4.3 (95% CI=2.1–9.0, *P*<0.001) and 13.3 (95% CI=6.5–27.4, *P*<0.001) times more likely to display positive attitudes towards people living with HIV. Similarly, safe practices related to safe sex were also observed among students with medium (OR=2.8, 95% CI=0.9–8.8, *P*=0.069) and high levels of knowledge (OR=1.9, 95% CI=0.6–6.2, *P*=0.284). More than three-quarters of students mentioned television and radio as major sources of information on HIV/AIDS.

**Conclusions:**

Despite adequate knowledge about HIV/AIDS among the school students, misconceptions about routes of transmission were found. Negative attitudes to HIV/AIDS and risky practices were also present. Educational programmes with specific interventions are recommended to increase KAPs and to prevent new HIV infections among students in Lao PDR.

## Introduction

In Lao PDR, HIV infection prevalence was estimated to be 0.1% among the adult population [1,2] in 2007, 1.1% among commercial sex workers (CSWs) [3] in 2001, and 5.6% among men who have sex with men (MSM) in the Vientiane capital [4] in 2007. Over time, the number of HIV-positive patients has rapidly increased. At the end of 2007, the official cumulative number of HIV-positive cases was 2400, of which 1523 were known to have developed AIDS and 775 to have died. Among people living with HIV (PLHIV) in Lao PDR, 58% were male and aged between 20 and 39 years old, and the major (85%) mode of transmission was heterosexual intercourse [1].

The prevalence of HIV infection in the adult population in Lao PDR was reported to be low compared to that of neighbouring countries [5]. However, there is no room for complacency. Geographical location of the country and several risky behaviours among its population may lead to a further spread of HIV across the country. Lao PDR is a developing country with open borders with countries that have higher HIV prevalence [6,7].

Immigration of different types of people, especially low-income groups, who come to Lao PDR to work in infrastructure development, such as road and dam construction, is very common. Another important issue is internal migration of people. This includes students who move to cities or urban areas after high school to continue their studies or to work [8]. These young people are away from their families for years at a time and very often visit CSWs for sex. This increases the possibility of spreading HIV between them, and thereby to the general population [9]. Although CSWs in Lao PDR are exposed to different activities relating to safe sex practices and are presumed to be well-versed about safe sex issues, very often these sex workers and their male clients engage in unsafe sex, mainly because of the influence of their male clients [6].

Although people of any age and gender are susceptible to HIV, young people aged 15 to 25 years are more at risk of contracting it. According to the World Health Organization and the Joint United Nations Program on HIV/AIDS, youths are much more prone to HIV infection as a result of a lack of correct health information, indulgence in risky behaviours, and lack of access to adequate reproductive health services. Every day 5,000 young people aged between 15 and 25 years in the world become infected with HIV, which translates into almost 2 million new infections per year. Young people face greater challenges and more exposure to risks, including economic exploitation, changing lifestyles, global, regional and national conflicts, and the spread of sexually transmitted infections and HIV/AIDS [10]. Any intervention targeted at young men before they are sexually active could prevent new HIV infections.

In this study, we focused only on male students because we believe that HIV-related risky behaviours are more prevalent among males than females. This is due to men dominance over women during sexual activities. In Lao PDR, women are often hesitant to answer questions related to sexual activity and have comparatively less opportunities to mix with wider society. Socio-economic issues associated with poverty, including limited access to high-quality health care; the exchange of sex for drugs, money, or to meet other needs; and higher levels of substance use can directly, or indirectly, increase HIV risk factors among women [11]. Men play a more active role during sexual activity, and very often it is men who deny protective measures, such as condoms, during sex. Moreover, men often influence women in decisions about safe sex practices.

Male-to-male sexual contact is another important issue and has been an important route of HIV-1 spread since HIV/AIDS was first identified some 30 years ago [12]. Reports from Thailand, Cambodia and Senegal suggested an unlinked epidemic pattern between general population HIV rates and those in MSM in countries with relatively low and declining HIV prevalence among heterosexual populations, but which have greater than 20% prevalence of MSM [13,14]. However, in recent years there has been an increase in HIV infection among the most vulnerable groups, especially MSM and migrant workers in Lao PDR [15]. Taking all of these factors into account, we decided to focus on men in order to achieve a greater impact of this study.

Increasing knowledge of HIV/AIDS can be a powerful means of fostering positive attitudes and building safe practices among populations. Hence, a clear understanding about knowledge, attitudes and practices (KAPs) among any population is very important for planning to control or prevent the spread of HIV. Information about HIV/AIDS-related KAPs has been reported by a number of researchers in different countries. Several studies have been conducted in Nigeria and Ghana to investigate KAP levels among their populations. These studies found that knowledge about the transmission of HIV was poor in senior high school students in Nigeria (31%, 14.4%, 9.1% and 8% of the students studied identified sexual intercourse, blood transfusion, mother to child (vertical) transmission and intravenous drug use, respectively). Only 7.1% identified all of the listed four modes of transmission of HIV whilst 0.7% of the students identified all of the listed preventive methods [16]. A survey among adolescents aged between 10 and 19 years in Ghana reported sexual abstinence (78.1%), condom use (72.7%), fidelity to partner (72.5%), not sharing needles (76.4%) and reducing sexual partners (56.7%) as important factors in preventing AIDS [17]. Nearly half of the participants from the school of Pune (India) believed that HIV can be contracted from toilet seats [18] and another study from Afghanistan reported that 53.2% of subjects believed that mosquito bites can transmit HIV [18,19]. Negative attitudes toward PLHIV and sexual behaviours were also reported by another study [18]. Although HIV/AIDS-related KAPs are reported in studies from other countries, there was no such information for school students in Lao PDR. Therefore, this study was conducted among unmarried male high school students of grade VII in the Lao PDR to determine their level of KAPs regarding HIV/AIDS.

## Methods

### Study design, sampling and procedure

This cross-sectional study was conducted among grade VII unmarried male students from nine high schools in six districts of Lao PDR in September 2010. Selected districts were chosen purposively from 11 districts in one province with the highest HIV prevalence. Out of a total 6.6 million PLHIV in Asia and the Pacific in 2001, nearly one million were living in this region. It has a long border with Thailand, which has the highest number of PLHIV in the region. This province also belongs to the lower Mekong region where one-sixth of PLHIV of Asia and the Pacific live [8]. We selected nine schools out of those six districts with a total of 2400 male students of grade VII. As there was no existing data on KAPs among school students in Lao PDR, we calculated the sample size based on the estimate that 50% of students may have enough knowledge on HIV/AIDS. Precision was set at 10%. For *P* value 0.05% and 80% power of the study, our expected sample size was 196 students. Fearing a high non-respondent rate because of the sensitivity of the issue, our aim was to recruit 300 respondents. To obtain our desired target of 300 participants, we decided to recruit a set number of students from each school by choosing every 8th student as our final respondent through systematic random sampling. Based on the observation and history from the school records, students who were less than 16 years old and mentally or physically disabled were excluded.

Before data collection began, students were briefed about the technical terminologies used in the questionnaire and were given guidance on how to fill out the form. The researcher to the selected students in the classroom then distributed the self-administered questionnaires during the 45 minutes break of their class. The students were informed about the purpose of the study and were assured that their responses would be treated confidentially. Respondents were also informed that their participation was entirely voluntary and that they were free to decline to answer any question that made them feel uncomfortable. Written informed consent was obtained from all of the students. Prior permission to conduct this study was obtained from the head master of the involved school. Moreover, this study was approved by the National Ethics Committee for Health Research (NECHR), Ministry of Health, Lao PDR.

### Questionnaire

The aim of the questionnaire was to obtain information on the level of HIV/AIDS-related KAPs of the respondents, as well as their sources of information on the issue. The questionnaire was developed by the researchers after consulting with experts in the relevant field. We also reviewed the questionnaire developed by the global school-based health survey for South Asian countries and Family Health International's questionnaire on HIV/AIDS prevention in developing countries during the questionnaire development [8,20]. Our final questionnaire included questions relating to HIV knowledge, attitudes toward PLHIV and sexual practices, in addition to socio-demographic information. The questionnaire was divided into four parts. Part I focused on the socio-demographic characteristics of the respondents, including age, ethnic groups, residence, and religion and their sources of information about HIV/AIDS. Part II contained 21 knowledge-related items, which again were subdivided into two sections, with questions relating to transmission, and prevention and control of HIV/AIDS. We included both positively and negatively framed questions to assess their knowledge, as well as their misperceptions, about HIV/AIDS. Part III comprised 18 questions on attitudes towards PLHIV, which again included both positively and negatively framed questions. Finally, Part IV comprised 12 questions about practices related to HIV/AIDS, including sexual behaviour and daily activities. Before data collection began, the questionnaire was piloted with 10 students, testing for clarity, feasibility and appropriateness for the students. The questionnaire was developed in English, then translated into Lao language before data collection and finally translated back into English.

### Statistical analyses

Data were entered into a spreadsheet and exported to Statistical Package for the Social Science^®^ (SPSS) for Windows, version 18.0 software (SPSS Inc., Illinois, USA) for analysis. Descriptive statistics were used to describe demographic characteristics and KAPs about HIV/AIDS. Numbers and percentages were used to present categorical data. Mean (±standard deviation, SD) was used for normally distributed continuous data, and median (interquartile range, IQR) for non-normal continuous data. Odds ratios (ORs) and 95% confidence intervals (CIs) were calculated though a logistic regression model to determine association levels of knowledge with attitudes and practices. All tests were two-tailed, and *P*<0.05 was considered significant.

To evaluate knowledge and attitude of the respondents, we asked them to answer “yes”, “no” or “do not know” to every knowledge- and attitude-related question. For practices, options were only “yes” or “no”. We assigned a score of 1 for a correct answer and 0 for a wrong answer for knowledge- and practice-related questions, and 1 for every positive answer in the attitude section and 0 for negative answers. The scores were then summed up to generate an overall score for each participant. Levels of KAPs were then re-categorized depending on their total, mean and median score. Accordingly, level of knowledge was categorized into “low” for respondents scored ≤ 50%, “moderate” for those scored between 51 and 74%, and “high” for those who scored≥75% [17]. The scores of attitudes and practices were categorized into two segments based on their mean and median score: those scoring less than mean scores for attitude were classified as “negative” and those scoring equal and more than mean scores were classified as “positive” attitudes. As data for practice was not normally distributed, we used median as the cut-off. Accordingly, those scoring less than median scores for practice were classified as “risky” practices, and those scoring equal and more than median scores were classified as “safe” practices.

## Results

### Socio-demographic characteristics

The mean age of the 300 participants was 17.9 years (SD±0.9), ranging from 16 to 19 years. Overall, 243 (81.0%) of the respondents were Laolum, and the majority (81.3%) were Buddhist. A high number of respondents were from district I (26.3%), and only 22 (7.3%) were from district VI (Table 1). All participants were living with parents and siblings.

**Table 1:** Socio-demographic characteristics of the study population (*N* **=**300)

Variable	Number	%
Age		
Mean±SD=17.9±0.9 years		
Range=16–19 years		
Ethnic group		
Laolum	243	81.0
Laosung	35	11.7
Laotheung	22	7.3
Religion		
Buddhist	244	81.3
Believe in spirit	56	18.7
Region (district)		
I	79	26.3
II	66	22.0
III	56	18.7
IV	50	16.7
V	27	9.0
VI	22	7.3

### Knowledge of HIV/AIDS

As illustrated in Table 2, all of the respondents had heard of HIV/AIDS and 91.3% had heard about people who had died of AIDS. Overall, the knowledge about route of transmission of HIV was high for some factors and relatively low for other factors. Accordingly, 97.7% of respondents knew that HIV can be transmitted through sexual intercourse. The majority of students were also aware that HIV can be transmitted through sharing needles or syringes, from mother to child and through blood transfusions. However, there was confusion about some routes of transmission. For example, only 61.0% of the participants correctly answered that “shaking hands” with PLHIV does not spread HIV. There was confusion about routes of transmission e.g. nearly half of respondents incorrectly thought that HIV could be transmitted by eating from the same plate, drinking from the same glass, wearing the same clothes and sharing the same toilet with PLHIV. Only a quarter of the respondents correctly answered that mosquitos do not transmit HIV.

**Table 2:** Knowledge regarding transmission and prevention of HIV/AIDS (*N* **=**300)

Questions with correct response	Number (%) reporting the correct responses
Knowledge about route of transmission	
HIV can be transmitted by sexual intercourse (Yes)	293 (97.7)
HIV can be transmitted from mother to child (Yes)	265 (88.3)
HIV can be transmitted by sharing needle or syringe (Yes)	276 (92.0)
HIV can be transmitted by blood transfusion (Yes)	222 (74.0)
HIV can be transmitted by shaking hand (No)	183 (61.0)
HIV can be transmitted by eating and drinking from the same plate or glass of an HIV-positive person (No)	178 (59.3)
HIV can be transmitted by wearing the same clothes of an HIV-positive person (No)	167 (55.7)
HIV can be transmitted by sharing a toilet with an HIV-positive person (No)	152 (50.7)
HIV can be transmitted through a mosquito bite (No)	77 (25.7)
Knowledge about prevention and control	
HIV can be prevented by not sharing needle or syringe (Yes)	274 (91.3)
HIV can be prevented by properly using condom during sexual intercourse (Yes)	268 (89.3)
HIV transmission can be avoided by remaining faithful to a single partner (Yes)	242 (80.7)
HIV transmission can be avoided by a blood test before marriage (Yes)	157 (52.3)

Table 2 also summarizes the knowledge of the students about prevention of HIV. A satisfactorily high level of knowledge was reported by students when they were asked questions such as: Can HIV be prevented by not sharing needle or syringe? Can using condoms during sexual intercourse protect against HIV infection? Can HIV be controlled by sticking to a single partner? However, in response to the question whether “HIV can be controlled by having a blood test before marriage” only 157 (52.3%) replied “yes”. Overall, respondents had a mean (±SD) score of knowledge of 13.6 (±3.6) from 21 knowledge-related questions. Accordingly, 46.3% were classified as having a high level of knowledge with a knowledge score of ≥75%, 31.3% as medium level of knowledge with 51% to 74% score and 22.4% as low level of knowledge with ≤50% score of knowledge regarding HIV/AIDS [17].

### Attitudes toward PLHIV

Overall attitudes of the high school students are illustrated in Table 3. The students exhibited positive attitudes to taking care of their HIV-positive relatives if they were ill and continuing friendships with HIV-positive friends. However, less than half of the students showed positive attitudes on issues such as: buying items from a HIV-positive shopkeeper or food seller; if an HIV-positive student should be allowed to continue her/his studying in school and if an HIV-positive teacher should be allowed to continue her/his teaching in school. Of the 18 questions that addressed attitudes toward PLHIV, the scores ranged from 3 to 18 (mean score=12.4, SD±3.3). Accordingly, 55.7% of students scored equal or more than the mean and were classified as having a positive attitude towards PLHIV. A total of 44.3% were classified as having a negative attitude toward PLHIV because they scored less than the mean.

**Table 3:** Attitudes towards people living with HIV/AIDS (*N* **=**300)

Questions with positive response	Number (%) reporting the specified responses
If one of your relative, who is HIV positive, becomes ill, would you be willing to care for her/him in your house or community? (Yes)	230 (76.7)
If your friend is HIV positive, would you continue your friendship with him/her? (Yes)	212 (70.7)
If a shopkeeper or food seller is HIV positive, would you buy items from him/her? (Yes)	146 (48.7)
If a student is HIV positive, she/he should be allowed to continue his/her studying in school? (Yes)	136 (45.3)
If a teacher is HIV positive, she/he should be allowed to continue his/her teaching in school? (Yes)	124 (41.3)

### Practices related to HIV/AIDS

Table 4 demonstrates that 94 (31.3%) of the school students had a history of sexual intercourse. The mean age (±SD) of their first sexual experience was 16.8 (±1.1) years with an age range of 14 to 19 years. Of these respondents, 14 (14.9%) had experience of sex with men in the form of oral, anal or both. Sixty-six (70.2%) students said they were using condoms during sex, only 29 (43.9%) were using condoms regularly when they had sex with casual partners and 35 (53.0%) used a condom during their most recent experience of sexual intercourse. A total of 56.4% of the school students reported safe practices, having an equal or greater than median score of 5.0, (IQR, 2.0–9.0) from 12 questions on safe sexual practices to prevent HIV/AIDS, and 43.6% were having risky practice, with less than the median score.

**Table 4:** Practices regarding HIV/AIDS

Variables	Number	%
Ever had sexual intercourse (*n*=300)	94	31.3
Ever had sex with men (*n*=94)	14	14.9
Ever use condom during sexual intercourse (*n*=94**)**	66	70.2
Use condom regularly during sexual intercourse with casual partner (*n*=66)	29	43.9
Use condom in the last sexual intercourse (*n*=66**)**	35	53.0
Ever drunk alcohol (*n*=94)	86	91.5
Had sex when under the influence of alcohol (*n*=94)	29	30.1

### Associations of knowledge with attitudes and practices

Binary logistic regression reports that level of knowledge significantly contributed to level of attitudes and practices as shown in Table 5. We found that respondents with medium and high levels of knowledge were likely to have proportionately positive attitudes (OR=4.3, 95% CI=2.1–9.0, *P*<0.001; and OR=13.3, 95% CI=6.5–27.4, *P*<0.001, respectively) and safe practices (OR=2.8, 95% CI=0.9–8.8, *P*=0.069; and OR=1.9, 95% CI=0.6–6.2, *P*=0.284, respectively).

**Table 5:** Association of knowledge about HIV with attitudes and practices related to HIV

	Attitudes^b^					Practices^c^				
		
Knowledge^a^	Positive %	Negative %	OR^d^	95% CI^e^	*P*	Safe %	Risky %	OR	95% CI	*P*
Low	7.8	40.6	1.0	Reference		13.2	26.8	1.0	Reference	
Medium	28.7	34.6	4.3	2.1–9.0	<0.001	54.7	39.0	2.8	0.9–8.8	0.069
High	63.5	24.8	13.3	6.5–27.4	<0.001	32.1	34.1	1.9	0.6–6.2	0.284

a Level of knowledge was defined as “Low” for score of 50% and less, Medium for 51 to 74%, and high for 75% and more [18].

b Level of attitude was defined as “positive” for those scoring ≥ mean, and “negative” for those scoring < mean.

c Level of practice was also defined as “Safe” for those scoring ≥ median, “Risky” for those scoring < median.

d OR=Odds ratio.

e CI=Confidence interval.

More than half (61.3%) of the respondents said that their most common source of information was the television, followed by the radio (25.3%). Less than one-fifth of the respondents were aware about HIV/AIDS from friends (7.7%), brochures (3.7%), newspapers (1.3%) and magazines (0.7%).

## Discussion

This is the first study on HIV/AIDS-related KAPs among high school students in Lao PDR. This study reports an average-level of KAPs relating to HIV/AIDS. However, misconceptions about the routes of transmission of HIV/AIDS remain. In addition, only just over half of the students showed positive attitudes towards PLHIV. Around a third of the students reported prior sexual experience including with a male partner.

Almost all survey respondents knew about HIV/AIDS and could correctly answer questions on HIV transmission and prevention, which indicates that students had a good basic awareness of the issue. Respondents also did moderately well in answering questions relating to the main routes of HIV transmission. Similar findings have also been reported in studies done in Afghanistan, Kazakhstan, Pakistan and China [19,21–23]. However, there was a lack of understanding about some important points of transmission of HIV, such as the belief that HIV can be transmitted by mosquito bites, along with shaking hands, sharing clothes, toilets and utensils with PLHIV. This indicates that students need more information and education about some points of routes of transmission. Again, similar misconceptions have been reported in other studies [19,22,23]. The majority of students knew that the use of condoms during sexual intercourse could prevent HIV. Similar findings were reported by previous studies conducted amongst Afghan university students [19], Turkish high school students [22] and Chinese undergraduate students [23] where almost half of the respondents were male.

Students exhibited mixed reactions to PLHIV. Whilst they displayed positive attitudes on most of the issues, they were reluctant to see an HIV-positive teacher continue to teach. This finding is not unique for Lao adolescents. Similar results were reported in several studies conducted in Ghana, China, Turkey and Iran [17,22–24]. The Iranian study, which was conducted in 2002 among 4641 second-grade high school students, reported that about half of the respondents (33%–46%) disagreed with PLHIV being able to enter schools, and said that they would not want to sit near PLHIV, or to shake hands with them. However, nearly half of the respondents also said they were eager to show compassion towards PLHIV [24]. This may be because students are empathetic toward PLHIV. However, they still fear that having close contact with them might put them at risk of contracting HIV. This is illustrated by a reported willingness to maintain a friendship or to be taught by PLHIV but not wanting to sit with them in the classroom. They might also believe that HIV is transmissible through items bought from an HIV-positive shopkeeper [19]. These attitudes are important to consider when developing strategies to respond to HIV.

Discriminating attitudes to PLHIV might be an obstacle for the efficient propagation of awareness programmes [19], and voluntary counselling and testing for HIV. Lao PDR has initiated a National Strategic and Action Plan on HIV/AIDS/STI for 2006 to 2010, which includes comprehensive guidance on efforts to respond to HIV and AIDS in the country. It identifies at-risk groups and corresponding strategies for targeted prevention; outlines strategies to enhance care and support for PLHIV; spells out policy, legal reform and advocacy measures; identifies measures to strengthen the capacity for surveillance and research; and outlines strategies for effective program management. This plan should carefully examine and include attitude issues with due importance. Many barriers, such as stigma and discriminatory attitudes [19], also need to be reduced.

PLHIV should be equally respected and valued in the society. The Lao Buddhist monks have been working to reduce social stigma towards PLHIV through their sermons, teaching in schools and being examples by providing spiritual care to PLHIV [25]. Adolescents need targeted counselling about safe practices by avoiding, for example, unprotected sexual relationships and exchange of syringes/needles. Risky sexual practices were also highlighted as another barrier for HIV prevention. One-third of high school students had a history of sexual intercourse, similar to the findings from other reports [21,23,26,27], and their first sexual contact was at 16 years of age [2,21,26,28–30]. Nearly 15% of surveyed student with sexual experiences said they had engaged in sex with men. This is an interesting finding in the conservative and religious Lao culture. Religion has a strong influence on personal as well as societal behaviour and practices of Lao people. Premarital and extramarital sex, as well as homosexuality, are discouraged by religious faiths in this population. Religious issues were also considered important and included in HIV/AIDS activities carried out by UNICEF in Lao PDR [7]. However, Lao PDR is also one among many countries that are trying to acknowledge these sexual behaviours in their governmental response.

Same-sex relationships have been legalized by the Lao government, which has enacted a special law. Male-to-male sex stigma is gradually decreasing, although it is not socially accepted yet [31]. This issue is still beyond the observation of many concerned authorities dealing with HIV/AIDS. Our results might offer important insights for them and stimulate necessary measures to be taken to prevent HIV transmission. Adolescents should be the focus of HIV/AIDS-related activities because this is the age when they become sexually active and are accessible through in-school education. Accurate HIV knowledge will support adolescents in making informed choices about their practices that may protect them from HIV transmission. Lao PDR is planning to introduce healthy living curricula for primary and middle school students and to incorporate this into the national school curriculum [8].

At present, various health educational programs are in effect at community-level including peer educator training. Sexual education in schools needs to be implemented soon. We found that less than half of the participants were using condoms consistently during sexual intercourse. This was consistent with other surveys in previous years [19,27,30]. In Lao culture, young and unmarried people buying condoms can be a sensitive issue. Adolescents might be embarrassed about buying condoms from shopkeepers out of fear that the shopkeepers might inform their guardians.

Although condoms are widely available in pharmacies, hotels, nightclubs and guest houses and are cheap to buy, affordability might still be a factor behind its inconsistent use by some young people. These findings were supported by a study, which identified unavailability of condoms as a major barrier to HIV prevention; only 34.2% of respondents said that they have access to condoms [19]. Some students said they were not comfortable using condoms during sex because they felt it might reduce their sexual pleasure. These misconceptions may increase the likelihood of HIV spread.

There were several limitations to the study. First, we restricted this study to only one province and did not include out-of-school adolescents. This limits the generalizability of the study findings to other provinces and to all adolescents of similar age. Second, whilst HIV knowledge is important, it may not be the primary factor in explaining HIV transmission among young people. Many people have adequate knowledge about HIV but do not act on it due to a wide variety of social, cultural and economic constraints. Future studies that investigate of all these possible constraints could help to improve our understanding of HIV transmission. Third, we used a three-point scale to assess attitudes that may not reflect respondents’ views with adequate precision. A five-point scale is usually a better choice in this case. However, fearing the ability of the school students to think in such wider scale, we hesitated to use a five-point scale. Finally, because of the self-administered questionnaire, social desirability bias may have occurred. However, the anonymity of the questionnaires hopefully encouraged students to be honest in their responses. Despite all of these limitations, we believe this study might be a reasonable source of information for researchers and policymakers.

## Conclusions

In conclusion, the surveyed high school students had moderate KAPs about HIV/AIDS. The study highlighted some misconceptions about HIV transmission, intolerant attitudes, stigma and discrimination towards PLHIV, and risky sexual practices, which need to be addressed. HIV/AIDS-related education programmes should include specific interventions to change practices, along with knowledge and attitudes. Future research involving nationally representative samples for both male and female, school-attending and out-of-school adolescents could contribute substantially to HIV/AIDS prevention.
